# A Real-Time De-Noising Algorithm for E-Noses in a Wireless Sensor Network

**DOI:** 10.3390/s90200895

**Published:** 2009-02-11

**Authors:** Jianfeng Qu, Yi Chai, Simon X. Yang

**Affiliations:** 1 College of Automation, Chongqing University, Chongqing, P.R. China 400030; E-Mails: sxbjq@163.com; chaiyi@cqu.edu.cn; 2 School of Engineering, University of Guelph, Guelph, ON, Canada, N1G 2W1

**Keywords:** Kalman filter, MOS gas sensor, noise reduction, data analysis

## Abstract

A wireless e-nose network system is developed for the special purpose of monitoring odorant gases and accurately estimating odor strength in and around livestock farms. This system is to simultaneously acquire accurate odor strength values remotely at various locations, where each node is an e-nose that includes four metal-oxide semiconductor (MOS) gas sensors. A modified Kalman filtering technique is proposed for collecting raw data and de-noising based on the output noise characteristics of those gas sensors. The measurement noise variance is obtained in real time by data analysis using the proposed slip windows average method. The optimal system noise variance of the filter is obtained by using the experiments data. The Kalman filter theory on how to acquire MOS gas sensors data is discussed. Simulation results demonstrate that the proposed method can adjust the Kalman filter parameters and significantly reduce the noise from the gas sensors.

## 1. Introduction

The environment is being affected more and more by the release of odorant pollutants in the atmosphere. These odors may discomfort the olfaction system and can even be harmful to human health. Electronic noses (e-noses) have been widely investigated [1,2], and are used for real-time environmental monitoring to prevent poison gas attacks by terrorists and gas leaks in chemical plants [3-5]. A single e-nose can only measures the odor strength at one location, and cannot evaluate the overall odor plume map around the monitored environment. The proposed wireless e-nose network system is able to collect remote odor data in real time and conduct further analysis for effective odor management.

In environment monitoring using a wireless e-nose network [3,5,6], accurate odor measurement is essential for many applications such as development of odor dispersion models and estimation of odor source location based on the odor data [7]. A wireless e-nose network system is composed of many e-nose nodes that are deployed in a monitoring region. These e-nose nodes are composed of an array of Metal-Oxide Semiconductor (MOS) gas sensors. The output signals from the MOS gas sensors contain not only gas signals, but also noise. The noise results in inaccuracies in analyzing data and estimating the odor strength. In a previous study, an e-nose consisted of a sensor array and an intelligent analysis system was developed, but the noise reduction of gas sensors was not well investigated [8,9].

The Kalman filtering algorithm is a recursive algorithm to solve the state estimation problems of known systems based on certain mathematical models and the observation of noisy measurements. Many modified filtering schemes have been developed to tackle the problems in various applications [10], e.g., a decentralized Kalman filtering algorithm to estimate collaborative information in wireless sensor networks [11], an adaptive Kalman filtering algorithm to reduce the noise for GPS and INS systems [12].

In this paper, a wireless e-nose prototype is developed to acquire MOS gas sensor output signals and send them to a remote server. A modified Kalman filtering technique is developed for improving the sensor sensitivity and precision of odor strength measurement. It can adapt in real time to adjust the measurement noise variance of the filter parameters. In addition, the optimal parameter of system noise variance is obtained by using the experimental data. Application of Kalman filter theory to the acquired MOS gas sensors data is discussed.

## 2. Hardware Development

**Figure 1.**
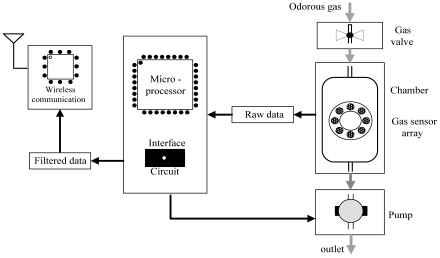
Block diagram of the e-nose prototype.

The block diagram of the proposed e-nose prototype is presented in Figure 1. It is mainly composed of two parts: the odorant gas measurement chamber unit, and the signal processing and wireless communication unit.

### 2.1. Development of the e-nose prototype

**Figure 2.**
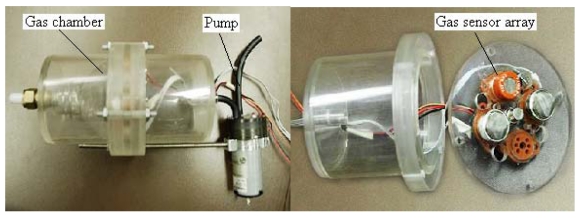
The gas chamber, pump and sensor array in the e-nose.

The odorant gas measurement chamber unit is shown in Figure 2. Based on previous extensive investigation and experiments, four MOS gas sensors (listed in Table 1) were adopted [13]. These four gas sensors can measure most of the major odorant gas compounds found in livestock farm odors. An electrical board is perforated with some holes and the four sensor pedestals are placed circularly; these pedestals have good compatibility, and can easily be replaced by different gas sensors. This electrical board is fixed on a plastic material chamber by using screws and nuts. A suction micro air pump is placed on the outlet of this chamber to ensure sufficient flow rate for the measurements.

**Figure 3.**
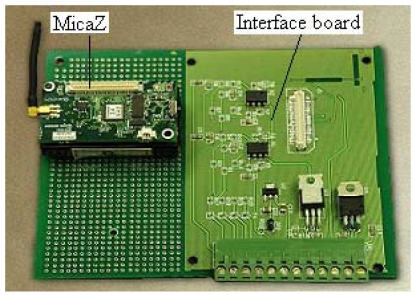
The interface board and the MicaZ for the e-nose.

The signal processing and wireless communication unit is shown in Figure 3, which is the brain of the e-nose prototype. The MicaZ node (from Crossbow Technology Inc., USA) is used and a voltage following circuit is situated on the data acquisition board. This unit is in charge of data acquisition, data processing and data transfer. The interface circuit uses only a voltage follower as a buffer between the sensor output and the A/D converter, which makes the system less sensitive to external disturbance. The MicaZ has advantages of the small physical size, low cost and low power consumption, making it ideal for this odor monitoring application. The MicaZ includes a processor and radio. The processor on the MicaZ primary consists of Atmegal-128L, which is in charge of data acquisition control, data processor control and data transfer control. The radio on the MicaZ primarily consists of a Chipcon CC2420, a basic 2,400 MHz ISM band transceiver compliant to IEEE 802.15.4/ZigBee protocol. Therefore, this unit of data acquisition, data processing, and data transfer ensures continuous data measurement.

### 2.2. Circuit design for the gas sensors

**Figure 4.**
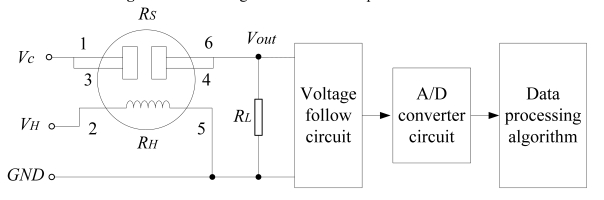
Block diagram of the data acquisition circuit.

A MOS gas sensor circuit and its interface diagram are shown in Figure 4, where *R_H_* is the gas sensor heater; R_s_ is the output resistance of the gas sensor, which changes with the variation of odor strength due to the presence of detectable odors. The voltage *V_out_* on the resistor *R_L_* will be changed as *R_S_* changes, the voltage *V_out_* can be measured, and then output resistance *R_s_* can be calculated as:

(1) $$R_{s}=\frac{Vc\times R_{L}}{V_{\mathrm{out}}}-R_{L}$$

The odor strength can be obtained from the table of sensor sensitivity characteristics curve by using the calculated *R_s_* value.

## 3. MOS gas sensor noise analysis

**Figure 5.**
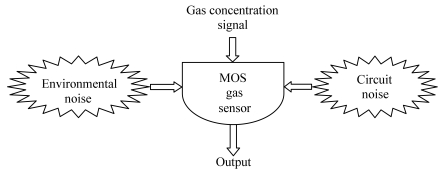
Block diagram of the inputs and outputs of an MOS gas sensor.

Noise unavoidably appears at all times in an odor sensing system. The two most common forms of noise are the circuit factor noise and environmental factor noise (see Figure 5).

### 3.1. Circuit factors noise

Circuit noise appears in the odor strength measurement process because the MOS gas sensors must work at the temperature of about 300°C, resulting in high resistor thermoelectric noise. Every semiconductor component of the interface circuit, such as voltage follower and regulated resistor, has its own circuit noise. Random movement of electrons and other charge carriers in resistors and semiconductors variation at random speed will result in random noise. Some noise also comes from factors related to the MOS gas sensors themselves. These factors include MOS gas sensor age, exposure to water and excess voltage, the bulk dissolution of surface atoms, mechanical wear and fatigue, self-heating, poisoning, and oxidation.

### 3.2. Environmental factors noise

In the actual odor strength measurement process, environmental factors such as ambient humidity, pressure variation and ambient temperature can all affect the output signals from the electronic gas sensors. Since MOS type gas sensors rely on the absorption and desorption of the odorant particles on their surface to generate signals, environmental factors can cause obvious changes in the response and the speed of the sensor response by altering the rate of the chemical reactions involved. The resistance of MOS gas sensors falls significantly as the humidity increases, but it will increase as the temperature increases. Furthermore, the impact on various gas sensors from environmental factors is not uniform, therefore, system parameters should be properly adjusted in the de-noise process as the environmental factors.

## 4. Kalman filter model for odor strength measurements

The Kalman filtering model is based on two sources of uncertainties: measurement noise introduced by the sensor and circuit noise, and the true strength variability; odor strength optimal estimation problem is modeled by a linear stochastic system. The system state vector *x_k_* and the measurement vector *y_k_* are described as:

(2) $$x_{k+1}=Ax_{k}+u_{k}+\omega_{k}=x_{k}+\omega_{k},$$

(3) $$y_{k}=Cx_{k}+\nu_{k},$$

where, *x_k_* is the system state vector; *y_k_* is the measurement vector; *u_k_* is the input vector (there is no input, *u_k_* =0); *ω_k_* is the process noise vector; and *v_k_* is the measurement noise vector. Pa rameter *A* is an identity matrix, which denotes the system matrix, and *C* is the measurement matrix, which transforms measurement voltage value to resistor value using Equation (1).

The noise covariance of *ω_k_* and *v_k_* are given as:

(4) $$E[\omega_{k}\omega_{k}^{T}]=Q,$$

(5) $$E[\nu_{k}\nu_{k}^{T}]=R_{k},$$

(6) $$E[\omega_{k}\nu_{k}^{T}]=0,$$

the *a priori* estimation error and estimation error variance are defined as:

(7) $$e_{K}^{-}=x_{k}-{\hat{x}}_{k}^{-},$$

(8) $$p_{k}^{-}=E[e_{k}^{-}{\left(e_{k}^{-}\right)}^{T}],$$

and, the *a posteriori* estimation error and estimation error variance are defined as:

(9) $$e_{K}^{+}=x_{k}-{\hat{x}}_{k}^{+},$$

(10) $$p_{k}^{+}=E[e_{k}^{+}{\left(e_{k}^{+}\right)}^{T}].$$

The system state prediction at step *k*+1 can be denoted by the *a posteriori* estimation at step *k* as:

(11) $${\hat{x}}_{k+1}^{-}={\hat{x}}_{k}^{+}.$$

The *a posteriori* estimation can be denoted by a linear combination of the *a priori* estimate and a weighted difference between actual measurement and the *a priori* estimate as [10]:

(12) $${\hat{x}}_{k+1}^{+}={\hat{x}}_{k+1}^{-}+k_{k+1}(y_{k+1}-C{\hat{x}}_{k+1}^{-}).$$

In practice, ${\hat{x}}_{k}^{+}$ is the strength estimation at step *k*; ${\hat{x}}_{k+1}^{-}$ is the predicted strength estimation at step; *k* + 1 ${\hat{x}}_{k+1}^{+}$ is the *a posteriori* strength estimation at step; *k* + 1; y_k+1_ is the actual strength measurement value at step *k*+1; the difference $\left(y_{k+1}-C{\hat{x}}_{k+1}^{-}\right)$ is called the measurement innovation, which reflects the discrepancy between the system state prediction and the actual measurement quantity; and the weighted value *k*+1 is the Kalman gain at step *k*+1. The Kalman gain *k_k_* is defined as:

(13) $$k_{k}=p_{k}^{-}{\left(p_{k}^{-}+R_{k}\right)}^{-1}=\frac{p_{k}^{-}}{p_{k}^{-}+R_{k}},$$

where $p_{k}^{-}$ and *R_k_* are from Equations (8) and (5), respectively.

The Kalman gain reflects the relationship between measurement and estimation. It indicates which one would be more reliable and should be “accepted” by the final estimation. The sensor is more reliable and the samples have lower variability, then the measurement error variance *R_k_* will be smaller, and the Kalman gain, which can be obtained from Equation (13), will be larger, because the Kalman gain is the weight factor of the measurement innovation in Equation (12); if the Kalman gain increases, then the weight factor of the measurement innovation will increase, and the *a posteriori* strength estimate quantity ${\hat{x}}_{k+1}^{+}$ will have more from the actual measurement value and less from the predicted value.

The *a posteriori* estimate error variance $p_{k}^{+}$ is defined as a function of the weight factor *k_k_* and the *a priori* estimation error variance. Thus the *a priori* and *posteriori* estimates $p_{k}^{-}$ and $p_{k}^{+}$ are defined as:

(14) $$p_{k}^{-}=E[(x_{k}-{\hat{x}}_{k}^{-}){\left(x_{k}-{\hat{x}}_{k}^{-}\right)}^{T}]=p_{k-1}^{+}+Q_{k-1},$$

(15) $$p_{k}^{+}=E[(x_{k}-{\hat{x}}_{k}^{+}){\left(x_{k}-{\hat{x}}_{k}^{+}\right)}^{T}]=(I-Ck_{k})p_{k}^{-}.$$

## 5. Modified Kalman filter

Tian *et al.* [8] analyzed the circuit and noise of MOS gas sensors in an e-nose; they concluded that the noise of these resistive type MOS gas sensors can be treated as Gaussian white noise plus some stronger low frequency direct current components. A standard Kalman filter solves the state estimation problems based on some certain assumptions in a system mathematical model, obtaining complete information about noise statistics as a Gaussian white noise; however, if there is uncertainty about the noise characteristics, the filter may not be robust enough. In this section, a modified filter algorithm based on the standard Kalman filter is proposed, which can adjust measurement noise covariance by using a slip windows average to reduce the noises, even if the sensor noise characteristics is unknown in advance.

### 5.1. Modified the measurement equation

The new odor strength measurement equation based on the noise analysis of MOS gas sensors can be modeled by:

(16) $$y_{k}=Cx_{k}+d_{k}+s_{k}.$$

where *d_k_* is the direct current noise component with the same frequency as the signal; *s_k_* is the white noise, *y_k_* is the measurement vector; and *C*is a measurement matrix.

A slip window average algorithm is proposed that is robust in estimating of the measurement average error. Given the window size *m*, the estimation of measurement average error is defined as:

(17) $${\hat{d}}_{k}=\frac{1}{m}\sum_{i=k-m+1}^{k}s_{i}.$$

From Equation (16), the white noise *s_k_* is given as *s_k_* = *y_k_* − *Cx_k_* − *d_k_*; if replace *x_k_* by the estimated value of odor strength *x̂*_k_, and replace *d_k_* by the measurement average error *d̂*_k_, then the measurement error can be defined as:

(18) $${ɛ}_{k}=y_{k}-C{\hat{x}}_{k}-{\hat{d}}_{k},$$

which includes all the measurement errors and white noise.

In Equation (16), replacing *d_k_* with the estimated measurement average error, *d̂*_k_ then have:

(19) $$y_{k}-{\hat{d}}_{k}=Cx_{k}+s_{k},$$

and, the new measurement vector *z_k_* can be defined as:

(20) $$z_{k}=y_{k}-{\hat{d}}_{k}.$$

From Equations (19) and (20), the new measurement equations can be expressed as a Gaussian white noise skplus the system state *x_k_*:

(21) $$z_{k}=Cx_{k}+s_{k}.$$

Plugging (20) into Equation (18), then have:

(22) $${ɛ}_{k}=z_{k}-C{\hat{x}}_{k}.$$

Replacing x̂*_k_* with the predicted strength estimation ${\hat{x}}_{k}^{-}$, then the new innovation *I_k_* is defined as:

(23) $$I_{k}=z_{k}-C{\hat{x}}_{k}^{-}.$$

### 5.2. Adaptive estimation of the measurement error covariance

The noise from the sensors and environment will shift dynamically during the odor strength measurement process. The measurement error variance should therefore also be adjusted dynamically in the actual filter implementation process, so an algorithm for adaptive estimation of the measurement error variance is proposed.

From Equation (6) *v_k_* is orthogonal to ω and ω is in parallel with every vector in *x_k_*, thus *v_k_* is orthogonal to every vector in, *x_k_* in particular to $x_{k}-{\hat{x}}_{k}^{-}$, that is:

(24) $$E(v_{k}(x_{k}-{\hat{x}}_{k}^{-}))=0.$$

From Equations (7), (8) and (24), the covariance of innovation can be obtained as:

(25) $$E(I_{k}I_{k}^{T})=E(v_{k}v_{k}^{T})+H_{k}E((x_{k}-{\hat{x}}_{k}^{-}){\left(x_{k}-{\hat{x}}_{k}^{-}\right)}^{T})H_{k}^{T},$$

(26) $$E(I_{k}I_{k}^{T})=E(v_{k}v_{k}^{T})+H_{k}{\hat{P}}_{k}^{-}H_{k}^{T},$$

where *P̂_k_* is the estimation of. *P_k_* Using the slip window average algorithm the covariance of innovation can be calculated as:

(27) $$E(I_{k}I_{k}^{T})=\frac{1}{n}\sum_{i=1}^{n}(z_{k}-H_{k}{\hat{x}}_{k}){\left(z_{k}-H_{k}{\hat{x}}_{k}\right)}^{T},$$

so the measurement noise covariance can be estimated as:

(28) $${\hat{R}}_{k}=E(v_{k}v_{k}^{T})=\frac{1}{n}\sum_{i=1}^{n}(z_{k}-H_{k}{\hat{x}}_{k}){\left(z_{k}-H_{k}{\hat{x}}_{k}\right)}^{T}-H_{k}{\hat{P}}_{k}^{-}H_{k}^{T}.$$

### 5.3. Error variance ratio factor λ

The measurement error variance represents how much noise the sensor is introduced into the measured strength from one measurement to the next one, and the process error covariance represents how much the true strength would vary from one measurement to the next one. In real-time estimation, the measurement error variance and process error covariance vary with the strength changes. Generally, determining the measurement noise variance in the actual implementation of the Kalman filter is possible. It can be determined by using the slip window algorithm proposed in this paper. Determining the system noise variance, however, is more difficult and complicated, as in practice the actual process value can only be estimated and it is impossible to obtain the accurate values. In the proposed algorithm, the process error covariance is estimated by using the error variance ratio factor, and the error variance ratio factor optimal range of each gas sensor is determined in the experiment, as given in Section 6.2. Thus, the error variance ratio can be defined as:

(29) $$\lambda =\frac{R_{k}}{Q_{k}}.$$

## 5.4. Modified Kalman filter algorithm

The modified Kalman filter reduced the noise in the strength measurement process by using feedback control. The filter estimates the strength from the odor strength at a previous time step and the sensor measurement with the noise component. The equations for the modified Kalman filter fall into two groups: time updating equations and measurement updating equations. The time updating equations are responsible for projecting forward the time, obtaining an *a priori* estimation and error covariance estimation of the next time step. The measurement updating equations are responsible for the feedback, incorporating a new measurement into the *a priori* strength estimation, in order to obtain an improved *a posteriori* estimation.

The time updating equations from time step *k−1* to step *k*, the previous state ${\hat{x}}_{k-1}^{+}$ and previous error covariance estimates $p_{k}^{-}$ are used to obtain the next time *a priori* estimations s ${\hat{x}}_{k}^{-}$ and $p_{k}^{-}$, which are given as:

(30) $${\hat{x}}_{k}^{-}={\hat{x}}_{k-1}^{+},$$

(31) $$p_{k}^{-}=p_{k-1}+Q_{k-1}.$$

The measurement updating equations incorporate a new observation *y_k_* into an *a priori* estimation ${\hat{x}}_{k}^{-}$ from the time updating equations to obtain an improved *a posteriori* estimation ${\hat{x}}_{k}^{+}$, which are given as:

(32) $${\hat{d}}_{k}=\frac{1}{j}\sum_{n=k-j+1}^{k}{ɛ}_{n},$$

(33) $${\hat{R}}_{k}=E(v_{k}v_{k}^{T})=\frac{1}{n}\sum_{i=1}^{n}(z_{k}-H_{k}{\hat{x}}_{k}){\left(z_{k}-H_{k}{\hat{x}}_{k}\right)}^{T}-H_{k}p_{k}^{-}H_{k}^{T},$$

(34) $$k_{k}=p_{k}^{-}{\left(p_{k}^{-}+R_{k}\right)}^{-1}=\frac{p_{k}^{-}}{p_{k}^{-}+R_{k}},$$

(35) $$p_{k}^{+}=(I-Ck_{k})p_{k}^{-},$$

(36) $${\hat{x}}_{k}^{+}={\hat{x}}_{k}^{-}+k_{k}(z_{k}-C{\hat{x}}_{k}^{-}),$$

(37) $$Q_{k}=\frac{R_{k}}{\lambda }.$$

The first step is to select an initial *a priori* value ${\hat{x}}_{0}^{+}$ and the error variance. *p_0_* The selection of these values has no constraints, since the filter will converge to an appropriate value automatically; however, if they are chosen in the dynamic range of the expected odor strength then the errors will converge rapidly.

Subsequently, the sensor performs a strength measurement to obtain *y_k_*, using the proposed slip window average method to estimate the direct current components *d̂_k_* and. *R̂_k_* The next step involves taking those estimations to compute the Kalman gain, *k_k_ a posteriori* error variance $p_{k}^{+}$ and *a posteriori* estimation ${\hat{x}}_{k}^{+}$. The following step is to calculate the system error variance *Q_k_* using the measurement variance and variance ratio factor. A loop cycle of the modified Kalman filter algorithm is thus finished. The filter will use the new *a posteriori* estimation and system variance in the time updating equations.

## 6. Results

The developed e-nose prototype was used in a livestock research farm at the University of Guelph in Canada. In the experiments, the four MOS gas sensors in Table 1 were installed in every e-nose node. In addition, the continuous heat regime was used in the experiments. By running the e-nose prototype to collect odor data for approximately 500 minutes in this period, each sensor acquired 500 simultaneous odor measurements data. A laptop computer running Matlab 7.0 software was used to process the data and to determine the optimal error variance ratio factor. These optimal values were then used in the noise reduction for the e-nose gas sensors.

### 6.1. Determination of variance ratio factor

A series of experiments were conducted with different values of *λ* in the de-noising process, in order to ascertain optimal range of this parameter. For simplicity without losing generality, the parameter determination for Sensor 1 is used as an example to exlpain the process. In the following figures, the thin line indicates the measured raw data, and the thick lines indicate the filtered data.

**Figure 6.**
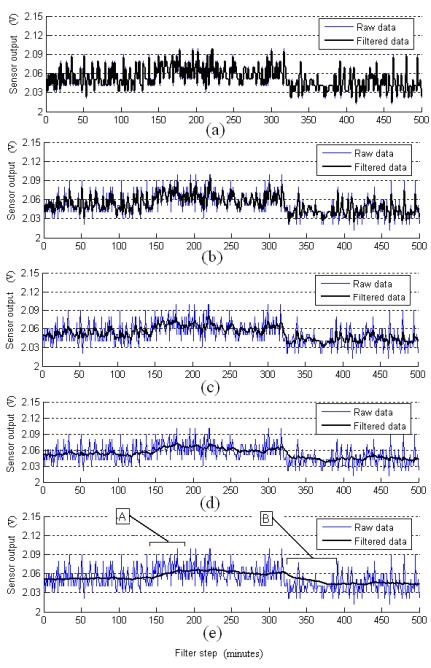
Filter results at different variance ratio factors *λ*. (a) *λ*=0.1, the filtered result fluctuates like the raw data; (b), *λ*=1 the filtered result is still fluctuating in some sections; (c) *λ*=10, the filtered result is still fluctuating in some sections; (d) *λ*=100, the filter result is robust for all the filtered ranges and sensitive to the changes in odor strength; (e) ***λ*** =1,000, a lag phenomenon appears in sections A and B in the filtered result.

As shown in Figure 6(a), the filtered data fluctuate tempestuously like the raw data when *λ* =0.1; the filter result is inaccurate, it cannot be used for actual real-time estimation of odor strength. The filtered results are shown in Figure 6(b) when *λ*=1 and in Figure 6(c) when *λ*=10; the filtered data is significantly smoother than the raw data, nevertheless, in some sections the filtered data still fluctuate sharply.

The filtered result when the optimal variance ratio *λ* increases from the range of approximately 100 is shown in Figure 6(d). It shows that the filter is effective, and the noise is reduced by using the filter. The performance of the filter is improved to an acceptable level when *λ* =100, and the parameter *λ* can be continued increasing to ascertain its optimal range. As shown in Figure 6(e), when *λ* increases to 1,000, the filter performance is more effective and the filtered line is smoother; however, in sections A and B in the filtered data line, the filtered line is lagged behind the true changes; the filtered line cannot denote the dynamic characteristics of odor strength changes.

Continuing the simulations using various *λ* values (including 50, 100, 150, 200, and 250) for the collection data, the results with all of these values were observed. The optimal parameter value of the variance ratio factor for the gas sensor 1 is 100. Fluctuations will appear tempestuously when the parameter value is too small, and lags will appear in the filtered line when the parameter is too large. Furthermore, gas sensor outputs will change with the sensors type, service time and manufacture.

For a more accurate result, the measurement error variance and the ratio need to be properly tuned. The optimal variance ratio *λ* for the four sensors in the e-nose prototype is obtained by the experiments, which are listed in Table 2.

### 6.2. Filtered results of the four gas sensors

**Figure 7.**
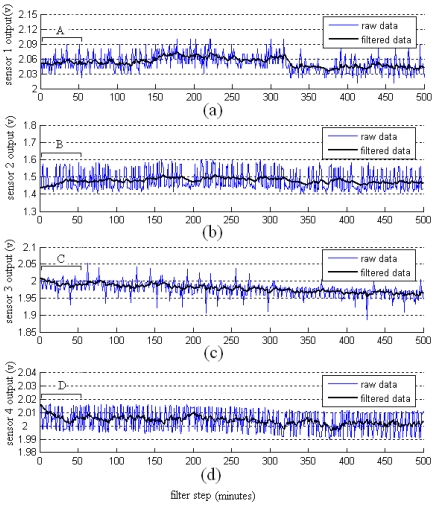
Filter results of the four sensors. (a) Sensor 1 at variance ratio factor *λ*=100; (b) Sensor 2 at variance ratio factor *λ*=70; (c) Sensor 3 at variance ratio factor *λ*=65; (d) Sensor 4 at variance ratio factor *λ*=120.

The noise of the gas sensors is reduced by using the proposed adaptive filtering technique. The results are shown in Figure 7. In the initialization transient periods (sections A, B, C, D in the figure), the filtered lines lag behind the raw data lines; meanwhile, the algorithm adjusts the parameters of the filters. After completing the adjustment to the parameter, the filters reduce the noise of the raw data, and estimate the optimal odor strength data. With the filter time prolonged, the parameter of the filter will adjust to the optimal value.

### 6.3. Comparison to the conventional Kalman filter algorithm

**Figure 8.**
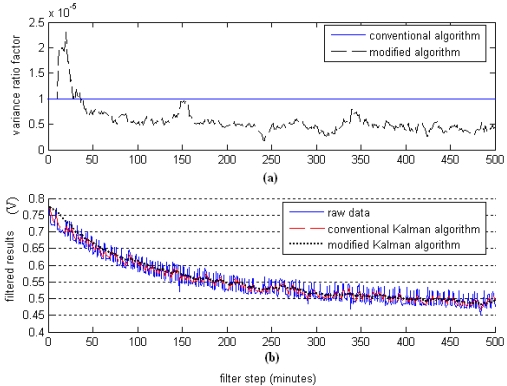
Comparison results between the conventional and the modified Kalman filter algorithm. (a) Estimated value of measurement noise variance; (b) The filtered results.

A standard Kalman filter solves the noise reduction problems based on assumptions that the measurement noise variance is a determined value; however, the measurement noise variance will fluctuate constantly with the filter step prolonged. Therefore, it is improper to assume that the measurement noise variance is a determined value. The measurement noise variances are shown in Figure 8(a). The filtered results by using the modified Kalman filter algorithm and the conventional Kalman filter algorithm are shown in Figure 8(b). Compared to the conventional Kalman filter algorithm, the modified one can estimate the measurement noise variance adaptively. Moreover, except for the initialization transient periods, the filtered results show considerable improvement in comparison to those using the conventional methods.

## 7. Conclusions

In this paper, a wireless e-nose prototype for a wireless sensor network that can accurately measure odorant gases and estimate odor strengths has been designed and implemented. The advantages of the e-nose prototype are its light weight, very small size, and flexibility in applications. Four commercial gas sensors are used. An interface circuit and a MicaZ are used for data acquisition, data analysis, and data transfer. Using the developed wireless e-nose network, remote and real-time odor measurements become possible.

Based on the output noise from gas sensor circuits, a real-time odor strength estimation model and a modified Kalman filter algorithm are proposed, which can improve the prediction capability and the accuracy of measurement. Using the proposed model and algorithm, the direct current component and Gaussian white noise are reduced considerably at the sensor outputs. In addition, even if the sensor noise characteristics is unknown in advance, the variance of measurement error can be changed adaptively. The experiments demonstrate that the modified Kalman algorithm is effective in the measurement of real-time odor strength of livestock farms odors.

## Figures and Tables

**Table 1.** Sensors used for the e-nose prototype.

***Sensors***	*Sensor 1*	*Sensor 2*	*Sensor 3*	*Sensor 4*
***Sensitive to gases***	Butane	Hydrogen-sulfide	Amine compounds	Air contaminants
***Concentration range***	2K-5K ppm	5-100 ppm	30-300 ppm	1-30 ppm of H2

**Table 2.** The optimal variance ratio factor. *λ* for sensors in the e-nose.

***Name***	*Sensor 1*	*Sensor 2*	*Sensor 3*	*Sensor 4*
*λ*	100	70	65	120
